# Postponing Distillability Sudden Death in a Correlated Dephasing Channel

**DOI:** 10.3390/e22080827

**Published:** 2020-07-29

**Authors:** Guanghao Xue, Liang Qiu

**Affiliations:** School of Materials and Physics, China University of Mining and Technology, Xuzhou 221116, China; TS18150001A31@cumt.edu.cn

**Keywords:** distillability sudden death, a correlated quantum channel, bounded-entangled states, free-entangled states

## Abstract

We investigated the dynamics of a two-qutrit system in a correlated quantum channel. The partial correlations between consecutive actions of the channel can effectively postpone the phenomenon of distillability sudden death (DSD) and broaden the range of the time cutoff that indicates entanglement of the positive partial transpose states. Particularly, the negativity of the system will revive and DSD will disappear in the fully correlated channel.

## 1. Introduction

Quantum entanglement plays a crucial role in quantum information science. However, it is usually fragile and degrades due to the inevitable interaction between the quantum system and its surrounding environment. Yu and Eberly found the phenomenon that the decay of entanglement of two-qubit system could be faster than that of the coherence of a single qubit, and they called it entanglement sudden death (ESD) [1,2,3,4]. The finite-time disentanglement could seriously wreck the application of entangled states in quantum information and computation. Subsequently, ESD attracts great attention in many physical systems [5,6,7,8,9,10,11,12,13,14,15,16,17,18,19,20]. There are reports on the evidence of ESD in optical setups and atomic ensembles [21,22,23]. Some schemes to delay or avoid ESD have also been proposed [24,25,26,27,28].

When it comes to the higher dimensional bipartite systems, rather than qubit–qubit or qubit–qutrit system, the entangled states can be divided into free-entangled states and bound-entangled states [29,30]. The former can be distilled to pure entangled states by local operations and classical communications (LOCC), while the latter cannot. Different from the free-entangled states, the bound-entangled states are usually useless for quantum information processing even though they may activate the teleportation fidelity [31]. Song and Ali found that the free-entangled states could decay to the bound-entangled states in a finite time due to the interaction between the two-qutrit system and the environment [32,33,34]. In other words, the distillable state becomes non-distillable during the evolution of the system. This phenomenon is named distillability sudden death (DSD).

All the unwanted interactions between the quantum system and its surrounding environment are considered to be the source of noises. A quantum channel transforming the input state into the output state can usually characterize the noisy effect in most cases. There may be partial correlations between successive uses of a channel because the channel may retain partial memory about its actions on the sequences of quantum systems passing through it. The partial correlations could be used to enhance two-qubit quantum coherence [35], protect quantum Fisher information [36] and reduce the entropic uncertainty of two incompatible observables [37].

However, the previous studies about the partial correlations of the channel focus on qubit systems. Higher dimensional or qudit system could provide certain benefits in quantum information and quantum computation. High-dimensional entangled systems can offer significant advantages for the manipulation of information carriers [38,39,40]. More efficient use of communication channels can be realized by biphotonic qutrit–qutrit entanglement [41,42]. One can also realize more resourceful quantum information processing by using hybrid qudit quantum gates [43]. In most cases, quantum information tasks require high-dimensional bipartite entanglement, and thus, there are some proposals to protect it. In [44,45,46], the authors gave the proposals to protect negativity by using weak measurement and reversal or environment measurement. However, the proposals to protect negativity of two-qutrit system are still scarce, and as we know, the proposal to postpone DSD has not been put forward.

In this paper, we investigate the influence of the partial correlations between successive actions of the dephasing channel on the negativity and distillability. The partial correlations could obviously postpone the decay of negativity and the sudden death of distillability. Moreover, the range of the time cutoffs for the entanglement of the positive partial transpose (PPT) states can also be broadened by the partial correlations. The remainder of the paper is organized as follows. In Section 2, we briefly recall some preliminaries about realignment criterion, negativity and the correlated quantum channel. Subsequently, we investigate in detail the influence of the partial correlations of the correlated dephasing channel on negativity and DSD in Section 3. A conclusion and discussion is given in Section 4.

## 2. Preliminaries

We first recall the notion of realignment criterion, which is used to detect the bound-entangled states [47,48]. The bound-entangled states are non-distillable under LOCC because they have PPT, while the states that have negative partial transpose (NPT) are distillable. The criterion to detect bound-entangled states is not unique even for the qutrit-qutrit system. Fortunately, the realignment criterion can detect the bound-entangled states employed in this paper. The realignment for a given density matrix ρ is ${\left(\rho^{R}\right)}_{ij,kl}=\rho_{ik,jl}$. The realignment criterion is $∥\rho^{R}∥-1\leq 0$ for a separable state and $∥\rho^{R}∥-1>0$ for a PPT state, and thus the positive value of $∥\rho^{R}∥-1$ can imply the bound-entangled states. However, it is important to note that the realignment criterion cannot detect all bound-entangled states.

In order to investigate the time evolution of entanglement for a two-qutrit system, we should adopt an appropriate measure of entanglement. Negativity can be adopted to measure the entanglement of free-entangled states, i.e., it is a measure of NPT states [49]. Given a density matrix ρ, negativity is defined as

(1) $$N\left(\rho \right)=\frac{∥\rho^{T_{i}}∥-1}{2},$$

which equals the sum of the absolute value of negative eigenvalues of $\rho^{T_{i}}$. Here, $\rho^{T_{i}}$ is the partial transpose of ρ.

One should keep in mind that negativity cannot tell us its entangled or separated for a PPT state, which has zero negativity. In this paper, the time evolution of the realignment criterion is investigated after the negativity becomes zero so as to determine whether the state is bound-entangled. An initial NPT state (free-entangled state) that is distillable evolves into a PPT state (bound-entangled state) that is non-distillable in a finite time. That is to say, the system suffers a transition from the distillable state to the non-distillable one during the dynamic process, and the phenomenon is DSD.

If $\rho_{0}$ is chosen as the input state of the channel Λ, the output state of the system after it traverses the channel could be written as $\rho =\Lambda (\rho_{0})$, which is usually given as $\Lambda \left(\rho_{0}\right)=\sum_{i}K_{i}\rho_{0}K_{i}^{†}$ in the operator-sum representation. Here, the Kraus operators $\left\{K_{i}\right\}$ describe the actions of the channel Λ on $\rho_{0}$, and they satisfy the completely positive and trace preserving relation $\sum_{i}K_{i}^{†}K_{i}=I$ with *I* being the identity matrix.

The system considered consists of two non-interacting qutrits that traverses the dephasing noise successively. The time evolution of the density matrix for a two-qutrit system is

(2) $$\rho \left(t\right)=\sum_{i,j=0}^{8}p_{i,j}\left(E_{i}⊗E_{j}\right)\rho_{0}{\left(E_{i}⊗E_{j}\right)}^{†}.$$

In the equation, the operators are expressed as $E_{0}=I_{3},\;E_{1}=Y,\;E_{2}=Z,\;E_{3}=Y^{2},\;E_{4}=YZ$, $E_{5}=Y^{2}Z,\;E_{6}=YZ^{2},\;E_{7}=Y^{2}Z^{2},\;E_{8}=Z^{2}$. $I_{3}$ is the identity matrix of three dimensions, and

(3) $$Y=\left(\begin{array}{ccc} 0 & 1 & 0\\ 0 & 0 & 1\\ 1 & 0 & 0 \end{array}\right),Z=\left(\begin{array}{ccc} 1 & 0 & 0\\ 0 & \omega & 0\\ 0 & 0 & \omega^{2} \end{array}\right).$$

Here, ω=exp(2iπ/3).

For the case of the qutrits traversing the channel independently and identically, the joint probability is $p_{i,j}=p_{i}p_{j}$ with $p_{0}=\sqrt{1-p}$ and $p_{k}=\sqrt{p/8}$ (k=1,2,⋯,8). p=1−exp(−Γt/2) is the time-dependent decoherence parameter with Γ being the rate parameter. In this case, the dephasing channel has no memory effect on the history of its actions on the sequences of qutrits.

However, it is only a limiting case that the channel has independent actions on the qutrits, and the channel may retain partial memory when the qutrits traverses the channel within a short time interval [50,51,52]. One kind of such memory effect, which introduces the partial correlations between consecutive uses of the channel, was proposed by Macchiavello and Palma [50]. They gave the probability distribution function as

(4) $$p_{i,j}=p_{i}p_{j|i}$$

with $p_{j|i}=\left(1-\mu \right)p_{j}+\mu \delta_{ij}$. $\delta_{ij}=1$ for i=j, otherwise $\delta_{ij}=0$. The strength of the partial correlations is characterized by the parameter μ with 0≤μ≤1. μ=0 corresponds to the uncorrelated channel, while μ=1 describes the fully correlated channel.

## 3. Postponing Distillability Sudden Death

In this section, we consider the influence of partial correlations due to the partial memory effect of the channel on negativity and distillability of two-qutrit system.

**Figure 1:**
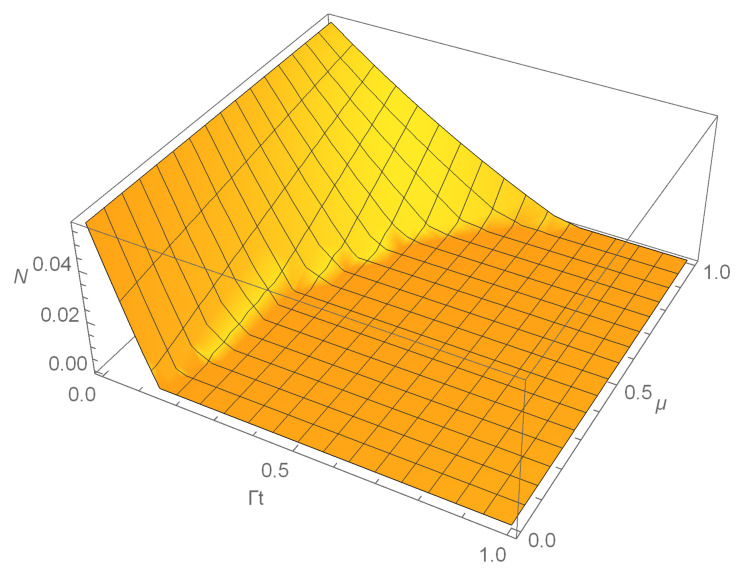
The dynamics of negativity of a two-qutrit system as functions of the time Γt and the strength of the partial correlations μ in the correlated channel. Here, the parameter of the initial state is fixed as α=4.3.

Let us assume the initial state of two-qutrit system is

(5) $$\rho_{\alpha }\left(0\right)=\frac{2}{7}|\Psi_{+}\rangle \langle \Psi_{+}|+\frac{\alpha }{7}\sigma_{+}+\frac{5-\alpha }{7}\sigma_{-}.$$

where $|\Psi_{+}\rangle =(|00\rangle +|11\rangle +|22\rangle )/\sqrt{3}$ is the maximally entangled state. $\sigma_{+}=(|01\rangle \langle 01|+\left|12\rangle \langle 12\right|+|20\rangle \langle 20|)/3$ and $\sigma_{-}=(|10\rangle \langle 10|+\left|21\rangle \langle 21\right|+|02\rangle \langle 02|)/3$ are the separated states. α is the parameter of the state with α∈[2,5]. For the separable states, 2≤α≤3, while for the bound-entangled and free-entangled states, 3<α≤4, 4<α≤5, respectively. Based on the Equations (2) and (4), the time evolution of the density matrix $\rho_{\alpha }\left(t\right)$ can be obtained. The eigenvalues of the partial transpose of $\rho_{\alpha }\left(t\right)$ can be straightforwardly determined, and we can investigate the dynamics of negativity. Because of its complexity, we do not give its analytical expression here. Instead, we perform numerical calculation and show in Figure 1 the influence of partial correlations on negativity.

**Figure 2:**
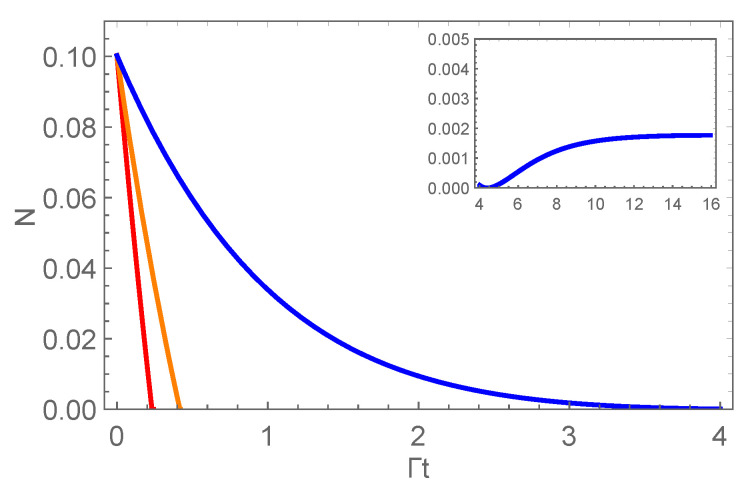
The time evolution of negativity of a two-qutrit system with μ=0,α=5 (red line), μ=0.5,α=5 (orange line) and μ=1.0,α=5 (blue line). The plot of negativity as a function of time is given in the inset with μ=1.0,α=5 to indicate the reviving of negativity.

From Figure 1, we can observe that negativity always decreases to zero in a finite time for a definite strength of partial correlations. Therefore, the decoherence effect of the dephasing channel is detrimental to negativity. On the other hand, Figure 1 tells us that the partial correlations can effectively postpone the decay of negativity. For a fixed time, negativity can be enhanced by the increasing of the strength of the partial correlations μ. The scaled time that negativity decays to zero is postponed by the correlated channel. These results can also be seen from Figure 2, where we plot the time evolution of negativity with different values of μ for the initial state of two-qutrit system being the maximally entangled state. The figure indicates that negativity decays to zero in a finite time except for the case of the fully correlated channel. The correlated channel prolongs the scaled time that there is negativity for two-qutrit system. Moreover, negativity first decreases to zero and then revives to a certain value for the fully correlated channel. The entanglement of two-qutrit system is partially preserved. The phenomenon that the initial quantum correlations of two-qubit system could be partially preserved has been reported before [53,54], and here, we focus on the preservation of entanglement for two-qutrit system.

**Figure 3:**
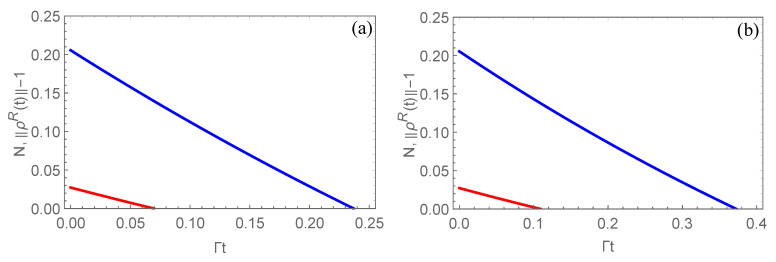
The time evolution of negativity (red line) and realignment criterion (blue line) in uncorrelated (**a**) and correlated channels with μ=0.5 (**b**). Here, the parameter of the initial state is fixed as α=4.3.

In the following, we demonstrate that the partial correlations can postpone DSD. Firstly, we should note that DSD could occur for a two-qutrit system, which can be found in Figure 3. From Figure 3a, the free-entangled state becomes the bound-entangled state at the time Γt=0.0697, and the realignment criterion indicates that there is entanglement for the PPT states in the range 0.0697≤Γt≤0.2397 when the channel is the uncorrelated one and α=4.3. If the correlated channel is considered, e.g., μ=0.5 in Figure 3b, the two-qutrit system loses its negativity at a later time Γt=0.1094, and in the range of 0.1094≤Γt≤0.3726, $∥\rho^{R}\left(t\right)-1∥$ is positive, indicating that PPT states are entangled. Obviously, the correlated channel postpones the occurrence of DSD, and the range of the time cutoffs for the existence of bound-entangled states is also broadened in the correlated channel. Through straightforward calculations, it is found that the correlated channel can postpone the DSD for other parameters of initial state, however, the ranges of the time cutoffs that there is entanglement for PPT states are reduced when α increases.

By applying local unitary operation $U=I_{3}⊗U_{3}$ with $U_{3}=\left|0\rangle \langle 1\right|+\left|1\rangle \langle 0\right|+\left|2\rangle \langle 2\right|$ on Equation (5), one can obtain the locally equivalent state $\sigma_{\alpha }\left(0\right)$

(6) $$\sigma_{\alpha }\left(0\right)=\frac{2}{7}|{\tilde{\Psi }}_{+}\rangle \langle {\tilde{\Psi }}_{+}|+\frac{\alpha }{7}{\tilde{\sigma }}_{+}+\frac{5-\alpha }{7}{\tilde{\sigma }}_{-}.$$

In the equation, $|{\tilde{\Psi }}_{+}\rangle =(|01\rangle +|10\rangle +|22\rangle )/\sqrt{3}$, ${\tilde{\sigma }}_{+}=(|00\rangle \langle 00|+\left|12\rangle \langle 12\right|+|21\rangle \langle 21|)/3$ and ${\tilde{\sigma }}_{-}=(|11\rangle \langle 11|+\left|20\rangle \langle 20\right|+|02\rangle \langle 02|)/3$ are respectively converted from $|\Psi_{+}\rangle$, $\sigma_{+}$ and $\sigma_{-}$ by the local unitary operation. According to the results given in [10,11], the local unitary transformations may affect the future trajectory of entanglement even if they have no influence on the static entanglement. Through numerical calculations, the correlated channel can still postpone the phenomenon of DSD and broaden the range of the time cutoffs that verify entanglement for PPT states. Particularly, negativity decays to zero and the state becomes the bound-entangled state at the time Γt=0.1030, which is slightly earlier than that of the state $\rho_{\alpha }\left(t\right)$. Furthermore, the time that the state $\sigma_{\alpha }\left(t\right)$ becomes non-distillable as well as the time that the realignment criterion cannot detect the entanglement turns to be earlier than those of the state $\rho_{\alpha }\left(t\right)$ with the increasing of the state parameter α.

## 4. Conclusions and Discussion

In this paper, we investigate the dynamics of negativity and realignment criterion of two qutrits that successively traverse the correlated dephasing channel. The probability that the same operation acts on the sequence of qutrits is used to characterize the partial correlations. We find that, for certain initial states, the partial correlations between the consecutive actions of the channel can effectively postpone the phenomenon of DSD and broaden the range of the time cutoffs that verify entanglement for the PPT states. Particularly, the negativity can revive, and thus, the partial correlations of the channel can avoid DSD of two-qutrit system in the fully correlated channel for the initially maximally entangled state. The results obtained in this paper are expected to be helpful in entanglement distillation protocols and some quantum communication tasks.

We remark that the results obtained in this paper are only valid for the states given in Equations (5) and (6) because the definitive and universal criterion for the separability or entanglement of the density matrix with dimensions greater than six is still absent [34]. For example, if the isotropic state

(7) $$\rho_{p}\left(0\right)=p|\Psi_{+}\rangle \langle \Psi_{+}|+\frac{1-p}{9}I_{9}$$

is chosen as the initial state, the time evolution of the density matrix $\rho_{p}\left(t\right)$ does not suffer DSD because the state has the property that its PPT region is always separable. Here, 0≤p≤1 and $I_{9}$ is the 9-dimensional identity matrix.
